# Validation and Diagnostic Performance of a CFD-Based Non-invasive Method for the Diagnosis of Aortic Coarctation

**DOI:** 10.3389/fninf.2020.613666

**Published:** 2020-12-09

**Authors:** Qiyang Lu, Weiyuan Lin, Ruichen Zhang, Rui Chen, Xiaoyu Wei, Tingyu Li, Zhicheng Du, Zhaofeng Xie, Zhuliang Yu, Xinzhou Xie, Hui Liu

**Affiliations:** ^1^College of Automation Science and Technology, South China University of Technology, Guangzhou, China; ^2^Department of Radiology, Guangdong Provincial People's Hospital, Guangdong Academy of Medical Sciences, Guangzhou, China; ^3^Department of Information Engineering, Northwestern Polytechnical University, Xi'an, China; ^4^Guangdong Cardiovascular Institute, Guangdong Provincial People's Hospital, Guangdong Academy of Medical Sciences, Guangzhou, China; ^5^Guangdong Key Laboratory of Medicine, Department of Medical Statistics and Epidemiology, Health Information Research Center, School of Public Health, Sun Yat-sen University, Guangzhou, China; ^6^School of Medicine, South China University of Technology, Guangzhou, China

**Keywords:** hydrodynamics, multidetector computed tomography angiography, non-invasive assessment, aortic coarctation, congenital heart disease

## Abstract

**Purpose:** The clinical diagnosis of aorta coarctation (CoA) constitutes a challenge, which is usually tackled by applying the peak systolic pressure gradient (PSPG) method. Recent advances in computational fluid dynamics (CFD) have suggested that multi-detector computed tomography angiography (MDCTA)-based CFD can serve as a non-invasive PSPG measurement. The aim of this study was to validate a new CFD method that does not require any medical examination data other than MDCTA images for the diagnosis of CoA.

**Materials and methods:** Our study included 65 pediatric patients (38 with CoA, and 27 without CoA). All patients underwent cardiac catheterization to confirm if they were suffering from CoA or any other congenital heart disease (CHD). A series of boundary conditions were specified and the simulated results were combined to obtain a stenosis pressure-flow curve. Subsequently, we built a prediction model and evaluated its predictive performance by considering the AUC of the ROC by 5-fold cross-validation.

**Results:** The proposed MDCTA-based CFD method exhibited a good predictive performance in both the training and test sets (average AUC: 0.948 vs. 0.958; average accuracies: 0.881 vs. 0.877). It also had a higher predictive accuracy compared with the non-invasive criteria presented in the European Society of Cardiology (ESC) guidelines (average accuracies: 0.877 vs. 0.539).

**Conclusion:** The new non-invasive CFD-based method presented in this work is a promising approach for the accurate diagnosis of CoA, and will likely benefit clinical decision-making.

## Introduction

The coarctation of the aorta (CoA) is a common congenital condition encountered in 6 − 10% of live births with congenital heart diseases (CHD) (Reller et al., 2008). Although CoA can occur as a solitary lesion, it is often associated with premature death and substantial late morbidity, including hypertension, heart failure, and premature coronary artery diseases (Toro-Salazar et al., 2002). Therefore, accurate diagnoses of CoA are important. In addition to anatomic evaluations, CoA can be clinically diagnosed by hemodynamic evaluations through cardiac catheterization (presently considered the standard method for its diagnosis and relative clinical decision-making). The specific diagnostic criterion of CoA is the occurrence of a peak systolic pressure gradient (PSPG) ≥20 mmHg (Rosenthal, 2001; Nielsen et al., 2005; Vogt et al., 2005; Menon et al., 2012a). Multi-detector computed tomography angiography (MDCTA) cannot be used to directly determine the occurrence of a PSPG; however, MDCTA-based computational fluid dynamics (CFD) can be employed to acquire hemodynamic information (e.g., pressure gradient) from coronary and cerebral arteries (Castro et al., 2006; Knight et al., 2010; Taylor et al., 2013). Still, the simulated pressure gradient depends on the applied boundary conditions, which cannot be directly determined from MDCTA images. Obtaining accurate boundary conditions is a persistent challenge for the clinical application of CFD-based methods, and should be overcome in order to perform unbiased CFD simulations. One approach to solve this problem would be to derive the boundary conditions from additional tests [e.g., transthoracic echocardiography (TTE) and 4D flow magnetic resonance imaging (MRI)] (Liu et al., 2016a; Xu et al., 2018; Zhu et al., 2018). Since CoA is a common CHD among pediatric patients, however, the need for additional tests is particularly inconvenient. Another approach would be to estimate the boundary conditions from several physiological models. This is similar to what is done when calculating coronary computed tomography angiography-derived fractional flow reserves (FFR-CT): several physiological models are used to estimate the approximate maximal hyperemia condition and obtain the correspondent boundary conditions. These physiological models reflect average behaviors and ignore the significant differences typically observed between pediatric patients, further degrading the accuracy of the CFD simulation results and limiting their real-life applicability.

The pressure drop occurring in correspondence of a coarctation can be approximate determined by using a common fluid dynamic equation (Gould, 1978; Banerjee et al., 2007):

(1) $$\begin{array}{r} \Delta \bar{p}=f\bar{Q}+s{\bar{Q}}^{2} \end{array}$$

where $\Delta \bar{p}$ is the mean pressure drop, f the viscous friction, s the expansion loss, and $\bar{Q}$ the mean flow rate. Aortic coarctation increases the viscous friction and causes the expansion loss of the stenosis section, enhancing the pressure drop: possibly, the first two parameters can be used to assess the hemodynamic severity of aortic coarctation. The proposed method can be used to obtain those two parameters by setting a series of boundary conditions comprised within a normal physiological range, and then performing a CFD simulation. Since the values of f and s in Equation 1 are almost independent of the flow rate and pressure, the results of the proposed method will not be affected with same parameter as boundary conditions. Compared to the general CFD method, the one proposed here does not require any medical examination data other than MDCTA images; hence, it represents a promising approach to achieve a non-invasive diagnosis of CoA.

The overall objective of the present study was to evaluate and validate the diagnostic performance of a novel CFD model developed from MDCTA imaging data for the diagnosis of CoA.

## Methods and Methods

### Ethic Approval

This retrospective study was approved by the local institutional review board following the ethical guidelines of the Declaration of Helsinki, and written informed consents were waived.

### Study Population

This study included a total of 65 subjects: patients with CoA (n = 38, median age = 9 months, ages ranging between 1 month and 14 years; 50% male) and others suspected of having CHD, but without evidence of CoA (n = 27; median age = 18 months, ages ranging between 2 months and 10 years; 59% male). All patients included in this study: (1) underwent MDCTA between February 2012 and September 2019; (2) underwent cardiac catheterization with recording of the aortic isthmus' PSPG <2 weeks before the time of the MDCTA; (3) were not subjected to any surgery or intervention before MDCTA. Patients with lesions in the branches of the aorta, or for whom we obtained poor-quality images, were excluded. More details about the study cohorts are presented in Table 1 and in the Supplementary Material of this paper (Supplementary Figure 1).

**Table 1:** Patients' information.

	**Patients with CoA**	**Patients without CoA**	***P-*value**
Number	38	27	
**Age**			0.183
Median	9 months	18 months	
Range	1 months−14 years	2 months−10 years	
**Gender**			0.614
Male	19 (50%)	16 (59%)	
**BSA**	0.462	0.569	0.193

*CoA, coarctation of the aorta; BSA, body surface area*.

### MDCTA and Cardiac Catheterization Protocol

MDCTA imaging was performed using an electrocardiographic-gated “step and shoot protocol” using a second-generation dual-source CT scanner (Somatom Definition Flash, Siemens Healthcare, Forchheim, Germany). A short-term sedation of the patients was achieved when necessary by administering them a 0.1 mg/ml oral chloral hydrate solution. Scans were performed in the cranio-caudal direction, from the thoracic inlet to the bottom of the heart. The MDCTA involved a gantry rotation time of 0.28 s, the use of a detector collimation (dimensions = 2 × 64 × 0.6 mm), and that of a CARE kV (with a weight-adapted grouping for the tube voltage and tube current). The acquisition window was grouped in the sequential mode at 35%−45% of the R-R interval. SAFIRE (Strength 3) was adopted as the iterative reconstruction algorithm, with I26 kernel, a slice thickness of 0.75 mm, and an increment of 0.5 mm. An iodinated contrast medium (Iopamidol, 300 mg I/ml, BRACCO, Italy) was injected intravenously (volume to body weight ratio of 1.5–2.0 ml/kg) for imaging, followed by 1.0 ml/kg body weight of a saline chaser injected at a rate of 1–2 ml/s. The acquisition delay was determined based on the time at which the contrast medium entered both ventricles.

The cardiac catheterization was performed using a Philips Allura Xper FD10 system (Philips Medical System, Best, the Netherlands). The PSPG was measured across the coarctation using the standard procedure, which inserted the catheter probe into the aortic isthmus of the patients, and recorded the peak systolic blood pressure of ascending and descending aorta.

### Boundary Conditions

**Figure 1:**
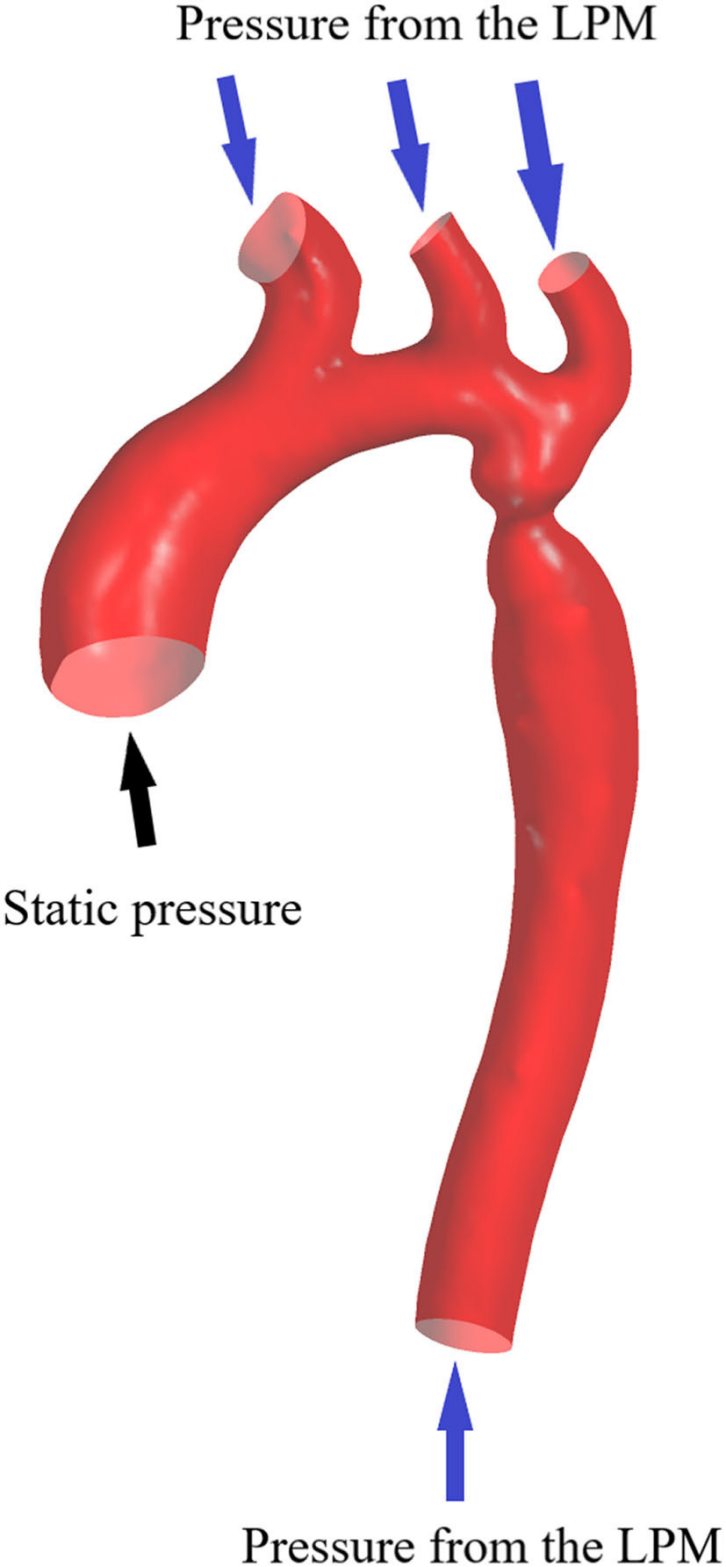
Boundary conditions. Geometry of the aorta with one inlet and four outlet boundaries.

**Figure 2:**
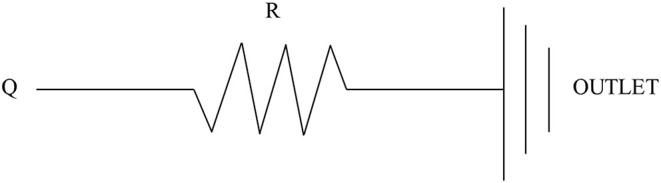
Scheme of the LPM. Outlet boundary condition: a lumped parameter model with only one resistance is coupled to each outlet.

The inflow and outflow boundaries are defined in Figure 1. The wall boundary was considered as a rigid vessel, and the flow domain was defined as a cavity of the reconstructed geometry. In the proposed method, a static pressure in the normal physiological range (80 mmHg) was mapped to the inlet of the CFD models. A lumped parameter model (LPM) with only one resistance was applied for each outlet, in order to confirm the outlet boundaries (Figure 2). A total resistance was allocated to each outlet according to their inverse diameters; then, the pressure value of each outlet was obtained by the LPM model (Murray, 1926; Taylor et al., 2013; Xie et al., 2018). The resistance was initially set to 9.6 mmHg·s /cm^3^, and subsequently reduced to 1/2, 1/3, 1/4, 1/5, and 1/6: the steady flow was simulated six times under six different total resistances for each case.

### Post-processing

The CFD simulation process is displayed in the Supplementary Material (method section, Supplementary Figure 2). The results of the CFD simulation were elaborated using CFD-Post 19.2 (ANSYS, Inc., Canonsburg, Pennsylvania, USA) and MATLAB (R2016a, the Math Works, Natick, MA). Only the stenosis sections with nearby branches are retained for further CFD analysis (as show in Figure 1), and the start and end of each lesion were defined by an experienced observer; then, the mean pressure in correspondence of the start and end sections and the mean flow rate across the coarctation were obtained from the simulation results. The pressure drop was defined as the pressure difference between the start and the end sections of the coarctation. After substituting the six steady flow simulation results into Equation 1, we obtained f and s through an iterative least squares estimation of the non-linear regression (George and Seber, 2003). Furthermore, the predictive parameters f and s obtained from the CFD simulation results were used to establish a combined diagnosis model by logistic regression.

(2) $$\begin{array}{r} P=\frac{1}{1+e^{-\left(af+bs+c\right)}} \end{array}$$

where *P* is the probability of the patients suffered CoA, f the viscous friction, s the expansion loss, and a, b, c are the coefficients obtained by logistic regression analysis using MedCalc Statistical Software version 19.0.7 (MedCalc Software bvba, Ostend, Belgium).The predictive performance of this model was evaluated on the AUC of the ROC curve by 5-fold cross-validation.

### Cross-Validation

**Figure 3:**
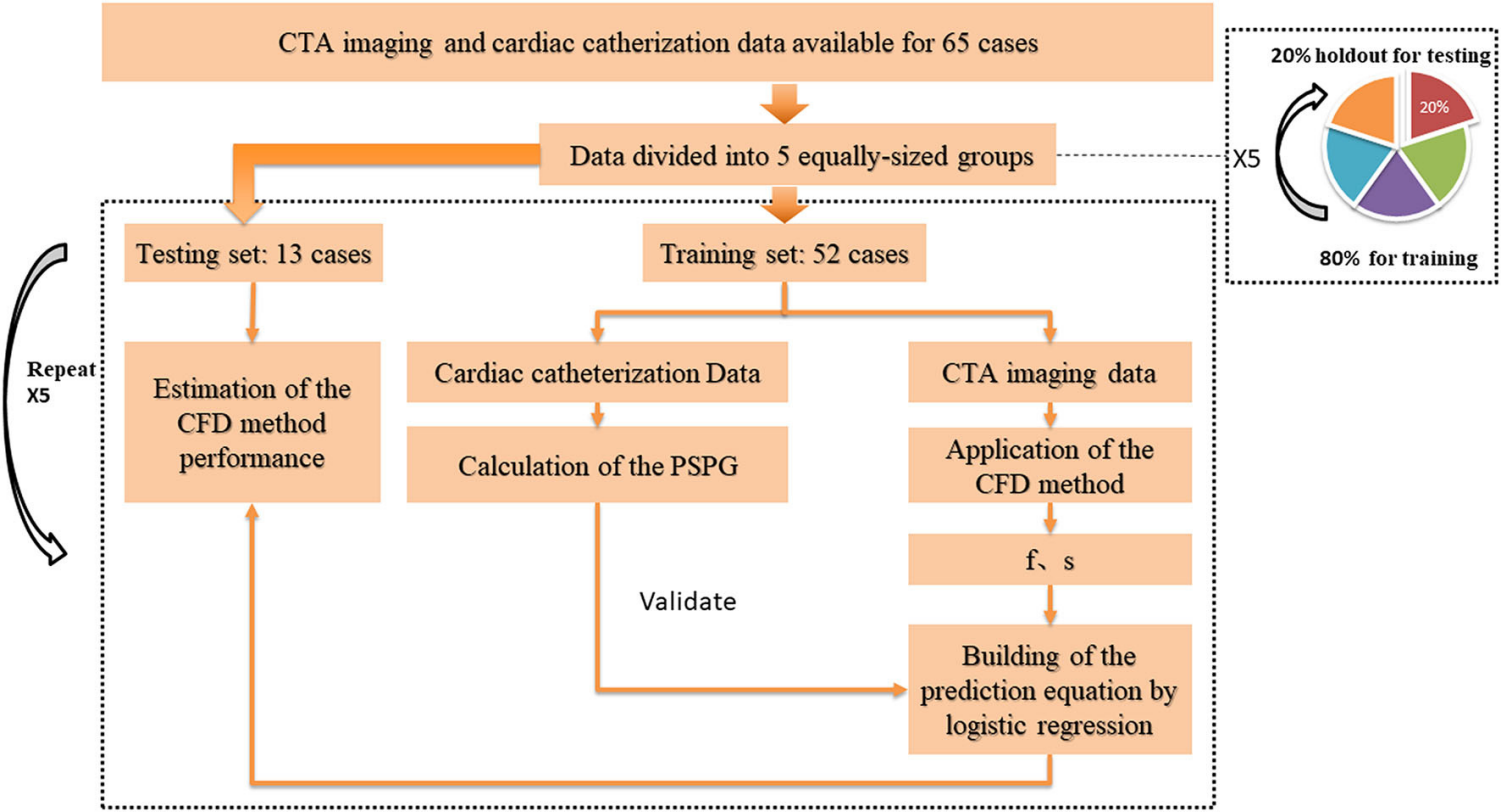
Cross-validation pathway. Five equally-sized groups were stratified so to have approximately the same proportion of genders, ages, and patients with CoA. One of them (20% of the data) was holdout for testing, while the others (80% of the data) were used as the training set. To estimate the CFD performance, we applied a 5-fold cross-validation procedure on all groups: each time, the CFD simulation was performed on a different training set. The parameters f and s, obtained from the simulation results, were used to build a prediction equation by using logistic regression, before testing the prediction model on the unseen test set.

To investigate and validate the diagnostic performance of the proposed method for CoA diagnosis, we randomly divided the study population into five non-overlapping groups having the same size. These five groups were stratified so to have approximately the same proportion of genders, ages, and patients with CoA. The diagnostic performance was then assessed by stratified 5-fold cross-validation (Figure 3). Compared to the conventional sample division method, the main advantages of the new approach are that: (1) it decreases the variance of the prediction error, (2) it maximizes the utilization of data from both the training and test groups, and (3) it avoids the testing of hypotheses suggested by arbitrarily split data. Overall, the proposed approach allows an unbiased estimate of the CFD performance in the diagnosis of CoA, removing uncertainties linked to the random division of one training group and one test group (Molinaro et al., 2005; Betancur et al., 2007; Kanamori et al., 2007; Motwani et al., 2010).

### Statistical Analysis

The continuous variables were expressed in the form of mean ± standard deviation (M ± SD). Normality was tested by the Kolmogorov-Smirnov method, and the variance homogeneity through the Levene test. The patient gender was analyzed using the Chi-square test, while the age and body surface area (BSA) data were analyzed through an independent samples *t*-test. The accuracy of the aorta reconstruction was validated by comparing the anatomic information with the Bland-Altman method. The correlations among the PSPG and CFD simulation results were evaluated based on a Spearman's rank correlation analysis. The diagnosis performance of the CFD method in training sets and test sets was evaluated using a ROC analysis and pairwise comparisons of the AUC according to DeLong et al. (Er et al., 1988). The diagnostic reference standard of the CoA is PSPG > 20 mmHg; therefore, we considered a cut-off value of 20 mmHg.

The statistical analyses were performed using the MedCalc Statistical Software version 19.0.7 (MedCalc Software bvba, Ostend, Belgium). All the tests were two-sided, and the results were considered statistically significant for *p* < 0.05.

## Results

**Figure 4:**
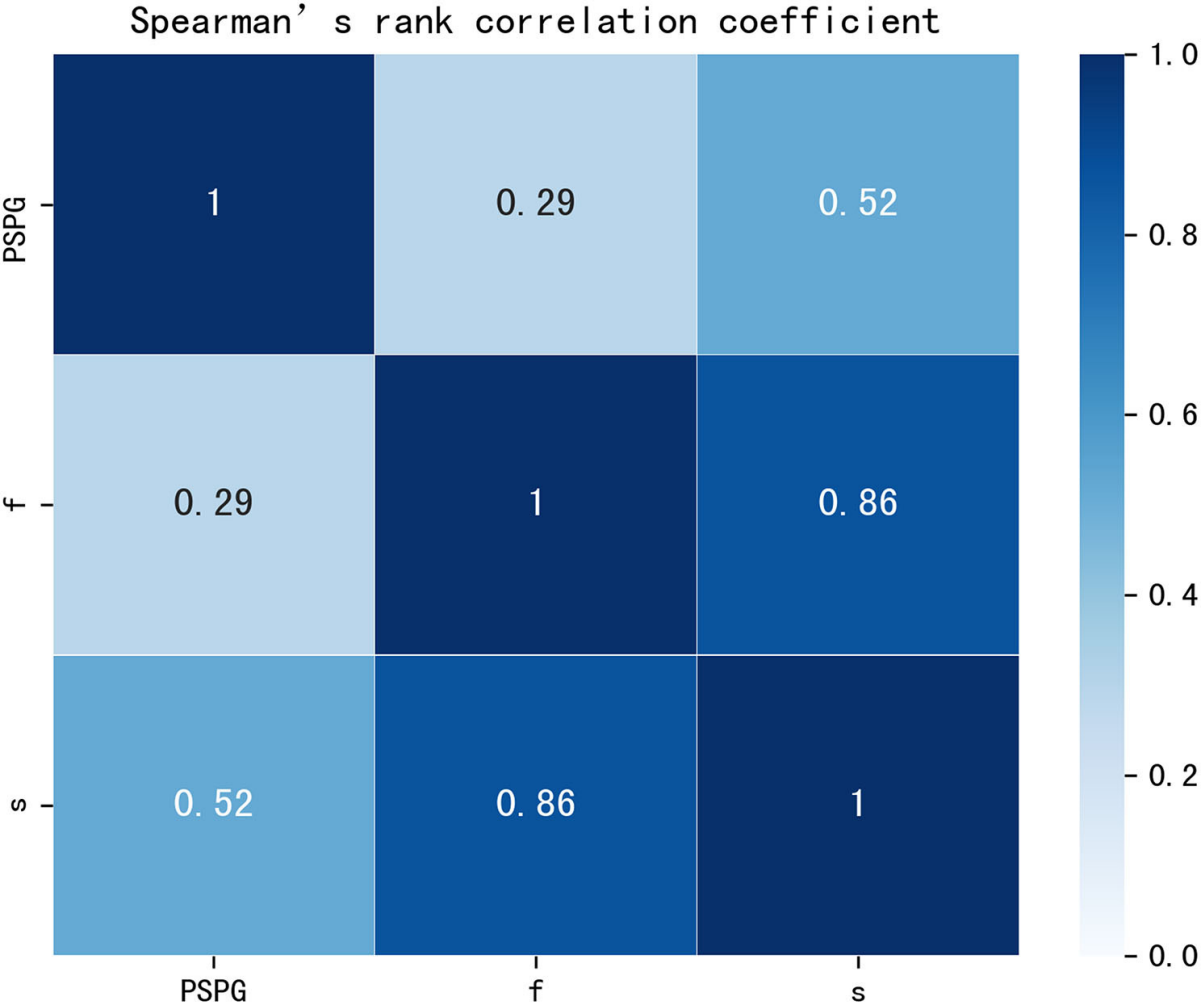
Rank correlation matrix among PSPG, f, and s. PSPG, f, and s were all positively correlated (*p* < 0.05). Lighter shades of blue correspond to lower correlation coefficients that the darker shades. PSPG, peak systolic pressure gradient; f, viscous friction in Equation 1; s, expansion loss in Equation 1.

No statistically significant differences we observed in terms of gender, age, and BSA (*p* = 0.183, *p* = 0.614, and *p* = 0.193, respectively) between patients with CoA and without CoA. Excellent agreement was observed between the diameters of the aorta measured through the CT workstation and those in the reconstructed models: the bias between the different datasets were of −0.024 ± 0.134 mm, −0.025 ± 0.141 mm, and −0.039 ± 0.129 mm, respectively (Supplementary Figure 3). A good correlation (rho = 0.861, *p* < 0.001) was noted between f and s, a moderate correlation (rho = 0.519, *p* < 0.001) was noted between PSPG and s, and a relatively low correlation (rho = 0.292, *p* < 0.005) was noted between PSPG and f (Figure 4).

### Performance of the CFD Method With Respect to the Training Set

**Figure 5:**
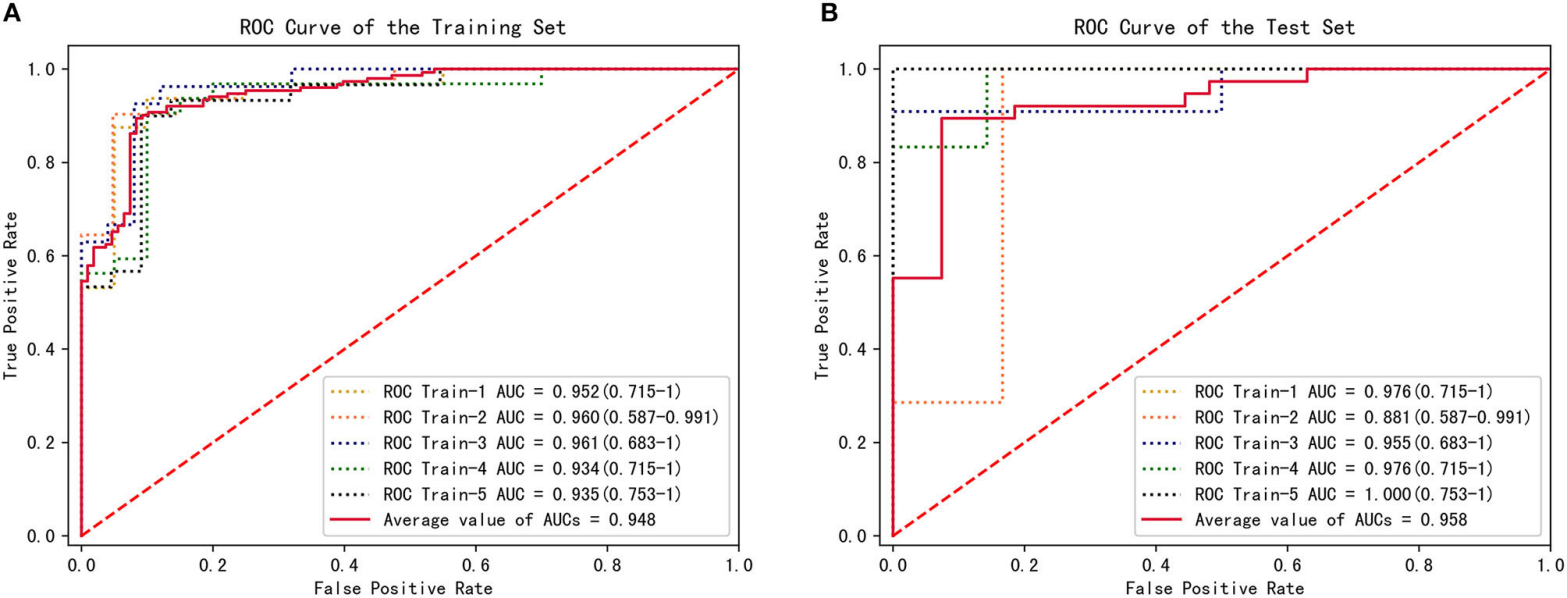
CFD performance for the diagnosis of CoA. **(A)** ROC for CoA diagnosis in the training set. **(B)** ROC for CoA diagnosis in the test set.

The parameters f and s in Equation 1 were obtained from the CFD simulation results; then, combined diagnosis models were established by logistic regression. The CoA diagnosis performances of these combined diagnosis models are shown in Figure 5A: all the training sets exhibited high AUCs (95.2, 96.0, 96.1, 93.4, 93.5%, respectively). The sensitivities, specificities, accuracy, and other details about these measurements are presented in Table 2. The sensitivities and specificities of the training sets exhibited high values (average values of 84.7 and 92.6%, respectively). The combined diagnosis models highlighted how 5, 4, 7, 7, and 8 out of 52 patients (in the case of 4, 3, 5, 5, and 6 false-negative patients, respectively) were misclassified in the five training sets. The average percentage of correctly classified patients was 88.1%.

**Table 2:** Diagnostic capacity of the CFD method in the training and testing sets.

****	**AUC (95% CI)**	**Specificities**	**Sensitivities**	**Accuracy**
**Training set (*****n*** **=** **52)**				
Train-1	0.952 (0.853–0.992)	0.95	0.875	0.904
Train-2	0.960 (0.866–0.995)	0.952	0.903	0.923
Train-3	0.961 (0.868–0.995)	0.92	0.815	0.865
Train-4	0.934 (0.830–0.984)	0.9	0.844	0.865
Train-5	0.935 (0.830–0.985)	0.909	0.8	0.846
Average value	0.948	0.926	0.847	0.881
**Testing set (*****n*** **=** **13)**				
Test-1	0.976 (0.715–1)	0.857	1	0.923
Test-2	0.881 (0.587–0.991)	0.833	0.714	0.769
Test-3	0.955 (0.683–1)	1	0.727	0.769
Test-4	0.976 (0.715–1)	1	0.833	0.923
Test-5	1 (0.753–1)	1	1	1
Average value	0.958	0.938	0.855	0.877

*The whole population was randomly divided into 5 equally-sized groups; then, the performance of the CFD method was assessed by a stratified 5-fold cross-validation*.

*AUC, area under the curve; CI, confidence interval; accuracy, % of cases correctly classified*.

### Performance of the CFD Method With Respect to the Test Sets

To estimate the performance of the CFD method, 5-fold cross-validation procedure was conducted on the five groups of data previously established. One combined diagnosis model was established for each training set, and then tested on the unseen test set. The performance of the combined diagnosis models with respect to the test sets is shown in Figure 5B. The five combined diagnosis models exhibited high AUCs (89.6, 91.7, 79.2, 97.9, 88.9%, respectively). The corresponding prediction models suggest that 12, 10, 10, 12, and 13 out of 13 patients were diagnosed correctly for each test set, respectively. Notably, 2, 3, and 1 patient(s) with CoA were misclassified (i.e., false negative) in the second, third, and fourth test sets, while no false negative cases were noted in first and fifth test sets. The performance of f and s in test sets are presented in Table 3.

**Table 3:** Diagnostic capacity of the f and s in the testing sets.

		**AUC (95% CI)**	**Specificities**	**Sensitivities**	**Accuracy**
Test-1	f	0.786 (0.479–0.957)	0.857	0.833	0.846
	s	0.881(0.587–0.991)	0.857	0.833	0.846
Test−2	f	0.714 (0.407–0.921)	0.833	0.571	0.692
	s	0.810(0.505–0.967)	0.833	0.714	0.769
Test-3	f	0.545 (0.257–0.813)	0.500	0.909	0.846
	s	0.773(0.465–0.951)	1	0.636	0.692
Test-4	f	0.571 (0.278–0.831)	0.429	0.833	0.615
	s	0.833(0.531–0.976)	0.857	0.833	0.846
Test-5	f	0.900 (0.610–0.995)	1	0.750	0.846
	s	1(0.753-1)	1	1	1
Average value	f	0.703	0.724	0.779	0.769
	s	0.859	0.909	0.803	0.831

*AUC, area under the curve; CI, confidence interval; accuracy, % of cases correctly classified; f, viscous friction in Equation 1; s, expansion loss in Equation 1*.

### Comparison With the ESC Guidelines Criteria

**Figure 6:**
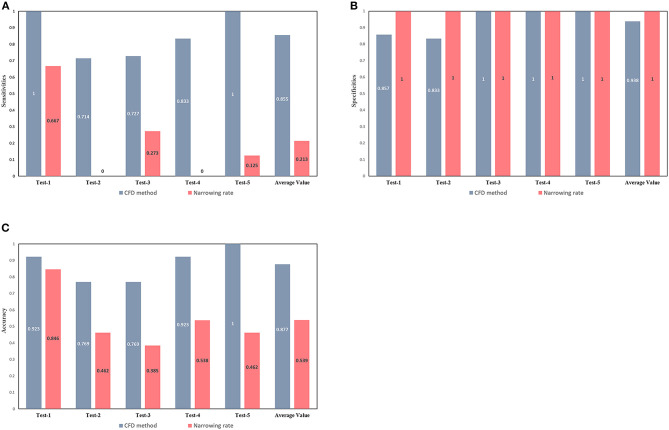
Comparison between the diagnostic capacity of the CFD method and of the ESC guidelines criteria. Sensitivities **(A)**, specificities **(B)**, and accuracies **(C)** of CoA diagnoses conducted by the CFD (gray-blue bars) and the narrowing rate (pink bars) methods. The latter method was applied according to current guidelines.

The European Society of Cardiology (ESC) guidelines indicate some non-invasive criteria for the determination of CoA. In particular, the Class II ESC recommends interventions on patients with a ≥50% aortic narrowing relative to the aortic diameter at the diaphragm level (observed by CMR, CT, or invasive angiography) (Kanamori et al., 2007). The narrowing rate (from the Class II ESC) and the results of the CFD method were compared to determine the occurrence of CoA (Figure 6). The narrowing rate is able to diagnose CoA with an average sensitivity of 0.213, an average specificity of one, and an average accuracy of 0.539 for the five test sets. Although the narrowing rate criteria exhibited excellent specificities in our test sets, the correspondent sensitivities were poor (average = 0.213). Remarkably, all patients with CoA in the second and fourth test sets were misclassified (false-negative) by applying the Class II ESC recommendation criteria.

## Discussion

CoA is associated with premature death and substantial late morbidity, including hypertension, heart failure, and premature coronary artery disease (Toro-Salazar et al., 2002). Therefore, accurate diagnoses of CoA are important. The observations conducted during the present study are relevant to the management of patients with suspected CoA. The viscous friction and the expansion loss of the aorta can be effectively used to classify pediatric patients with CoA, since they reflect the flow resistances causes by a given stenosis. Notably, these two parameters can be obtained by combined CFD simulations. The main advantages of the proposed method are that: 1) it allows a non-invasive diagnosis of CoA, 2) does not require extra medical examination data to establish boundary conditions, 3) is able to fully describe the pressure-flow relationship in a stenosis within a normal physiological range.

Current guidelines indicate that cardiac catheterization should be used to address specific anatomical and physiological questions, or before intervention. The reference standard for the diagnosis of CoA is a PSPG > 20 mmHg (Baumgartner et al., 2010). However, the standard procedure is invasive and costly; therefore, its clinical application should be limited to patients whose diagnosis is difficult or who need to be evaluated for subsequent intervention. TTE and 4D flow MRI can be applied to obtain the blood flow velocity; afterwards, the PSPG across the coarctation can be obtained by combining them in a simplified pressure estimation formula, which may result in an overestimation of the PSPG (Sakthi, 2010). Another approach is to apply the velocity values as boundary conditions, and then utilize the CFD method to acquire the PSPG across the coarctation. This method, however, needs additional tests (e.g., TTE or 4D flow MRI) and the process is complex: additional, non-contrast enhanced MRI, including 4D flow MRI, can be technically challenging, easily influenced by environmental noise and also limited by a relatively lower temporal resolution (Cibis et al., 2014; Khodarahmi, 2015). Meanwhile, the proposed method provides additional hemodynamic information and only requires the collection of MDCTA images.

The calculation of the hemodynamic parameters using CFD models developed from MDCTA imaging data is an attractive concept and potentially obviates the need for invasive angiography in pediatric patients suspected to have CoA (LaDisa et al., 2011). The present study focused on evaluating the diagnostic performance of a new MDCTA-based CFD model for the diagnosis of CoA. The results revealed that, in both the training and test sets, patients showed high AUCs and only a small number of them were misclassified. This indicates that the MDCTA-based CFD model has a high level of diagnostic efficiency. The misclassification of some patients in both the training and test sets could have derived from the use of actual simulation conditions in the present study. To reduce the computing time, we simulated a steady flow state and defined the pressure-flow relationship of a stenosis. A real pulsating blood flow, however, is inconsistent with the steady state flow assumption. To implement the pressure at the outlet boundary, previous studies have applied a lumped parameter model (Menon et al., 2012b; Liu et al., 2016b); still, the modeling of the aorta hemodynamics based on such model is inconsistent with real conditions (Kim et al., 2009, 2010), resulting in further biases during the CFD simulation.

A set of non-invasive criteria for the identification of patients with significant CoA and requiring intervention have been provided in the ESC Guidelines. Figure 6 shows how those non-invasive criteria performed poorly in our dataset and in that of another study (Astengo et al., 2017). Thus, relying on ESC recommendations for the identification of CoA patients might lead to under-diagnosis and the conservative treatment of many patients actually needing intervention. In fact, the narrowing rate criteria rely on simple morphological measurements, not taking into account any hemodynamical information (which may play a greater role in the development of a significant pressure gradient across the CoA). The proposed MDCTA-based CFD method can provide additional hemodynamic information and compared to the narrowing rate criteria, it shows an overall better diagnosis performance.

The present study has several limitations. First, we considered a relatively small sample size: we suggest to increase that in further studies. Moreover, the boundary conditions used for the CFD simulation were derived from the LPM model, which is inconsistent with real conditions. Still, the correspondent validation results suggest that the simulation error was negligible compared to that observed in another study (Kilner et al., 1993; Dwyer et al., 2009).

The results of the present study show that the proposed CFD model developed from MDCTA imaging data represents an accurate non-invasive method for the diagnosis of CoA, and which can be beneficial for clinical decision-making.

## Data Availability Statement

The data analyzed in this study is subject to the following licenses/restrictions: The authors do not have permission to share data. Requests to access these datasets should be directed to Qiyang Lu, 915081903@qq.com.

## Ethics Statement

The study was approved by the Clinical Ethics Committee of Guangdong Provincial People's Hospital. Written informed consent from the participants' legal guardian/next of kin was not required to participate in this study in accordance with the national legislation and the institutional requirements.

## Author Contributions

QL and WL drafted the manuscript and analyze the data. RZ provided the post-processing tools. RC, XW, TL, and ZY revised the manuscript. ZD provided the statistical analysis method. ZX provided the cardiac catheterization data. HL and XX provided the conception and design of the study. All authors contributed to the article and approved the submitted version.

## Conflict of Interest

The authors declare that the research was conducted in the absence of any commercial or financial relationships that could be construed as a potential conflict of interest. The reviewer QT declared a past co-authorship with the authors (XX, RZ, HL) to the handling editor.
