# Supplementary material for: Validation and Diagnostic Performance of a CFD-Based Non-invasive Method for the Diagnosis of Aortic Coarctation
Source: Front Neuroinform. 2020 Dec 9;14:613666. doi: 10.3389/fninf.2020.613666 (PMC7756015; doi:10.3389/fninf.2020.613666)
Supplement: Supplementary file 1 [file Table_1.DOCX]

**SUPPLEMENTARY DATA**

The following data have been provided as supporting material due to the limited space available in the main manuscript. Although they are not essential for the understanding of the paper, the authors felt that they could benefit readers and complement the main manuscript.

**Methods**

Online Figure 1 illustrates the enrollment and steps involved in this study.

***Construction of the aorta model and mesh generation***

Computational representations of the aorta were developed using the Mimics 19 software (Materialise, Leuven, Belgium), with the aim of facilitating volume visualization and the conversion of MDCTA imaging data into geometrically representative computer models. The aorta models were then discretized using an automatic mesh generation software (Ansys ICEM 19.2, ANSYS, Inc., Canonsburg, Pennsylvania, USA). The discretization procedure was customized based of the standard protocol for unstructured, formatted tetrahedral elements. Each developed model contained a total of 1,000,000–2,000,000 elements.

***CFD simulation***

The CFD simulation process is shown in Online Figure 2. The CFD simulation was performed using the Ansys FLUENT 19.2 software (Fluent Inc., Lebanon, New Hampshire, USA). All calculations were performed assuming the following (based on previous work): 1) the blood can be considered an incompressible, viscous, and Newtonian fluid; 2) the blood’s density (1050 Kg/m^3^) and viscosity were constant (0.0035 Pa∙s).

The following expressions of the Navier–Stokes equations were solved by Fluent automatic:

$\rho(\frac{du}{dt}+u\bullet\nabla u)=-\nabla P+\mu\nabla^{2}u+F$ (1)

$-\nabla\bullet u=0$ (2)

where ρ is the blood density, u the flow velocity, P the blood pressure, μ the blood viscosity, and F the body force (= 0).

The whole process consisted of 4 steps. First, we reconstructed a series of 3D models based on MDCTA images. Second, we discretized the 3D models into mesh files (each consisting of multiple mesh elements). Third, we defined the boundary conditions within normal physiological range values; in this step, we mapped a static pressure (within a normal physiological range) at the inlet of the CFD model. For each outlet, a lumped parameter model (LPM) with only one resistance was applied to confirm the outlet boundaries. Fourth, the mesh file, the inlet boundary conditions, and the outlet boundary conditions were imported into the Ansys Fluent software. The Fluent solver solved the dynamic fundamental equation for each mesh element and obtained the correspondent hemodynamics; subsequently, it integrated the hemodynamics into the whole flow field.

**Results**

The results of a consistency test between the reconstruction and anatomic information are show in Online Figure 4.

**Supplementary Figures:**

Online Figure 1 Enrollment and steps involved in the study


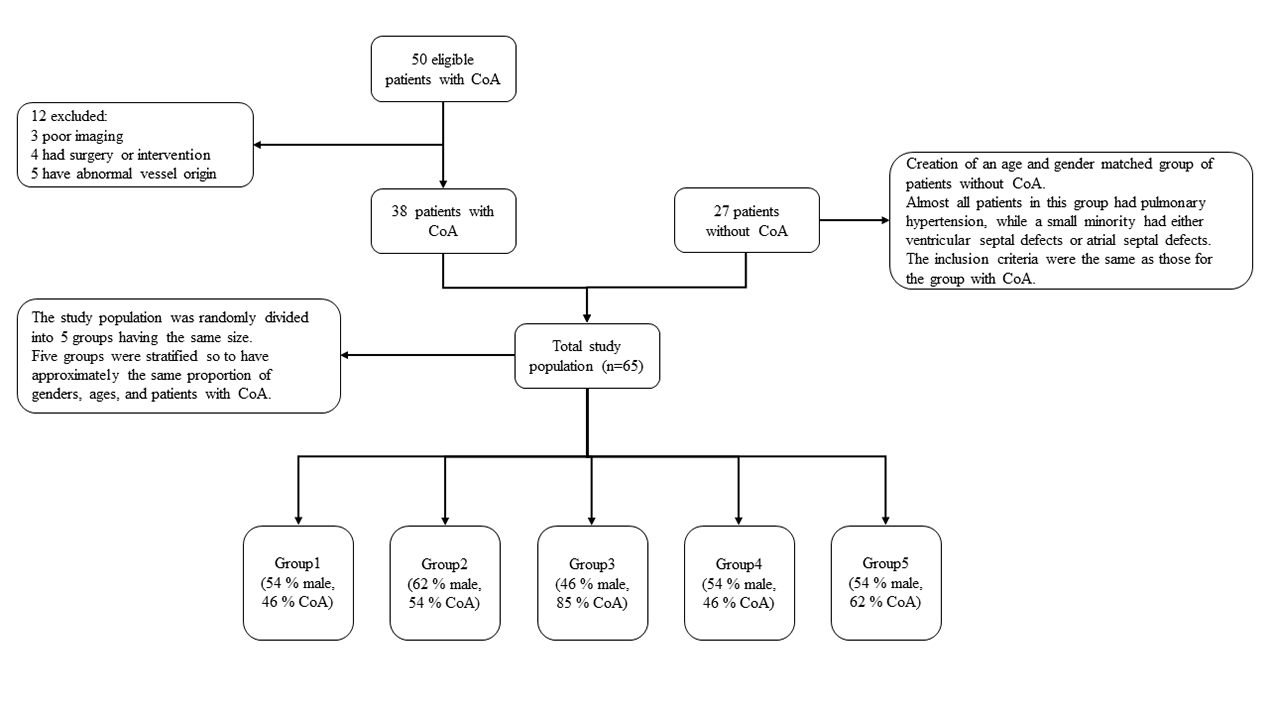


The patient group comprised of patients (n = 38) with CoA confirmed by cardiac catheterization. Patients with lesions in the branches of the aorta, or for whom we obtained poor-quality images, were excluded. The control group (n = 27) was age- and gender-matched with the patient group. The subjects in this group were patients suspected to have CHD but without evidence of CoA. The study population (n = 65) was randomly divided into 5 groups having the same size.

Online Figure 2 CFD Process


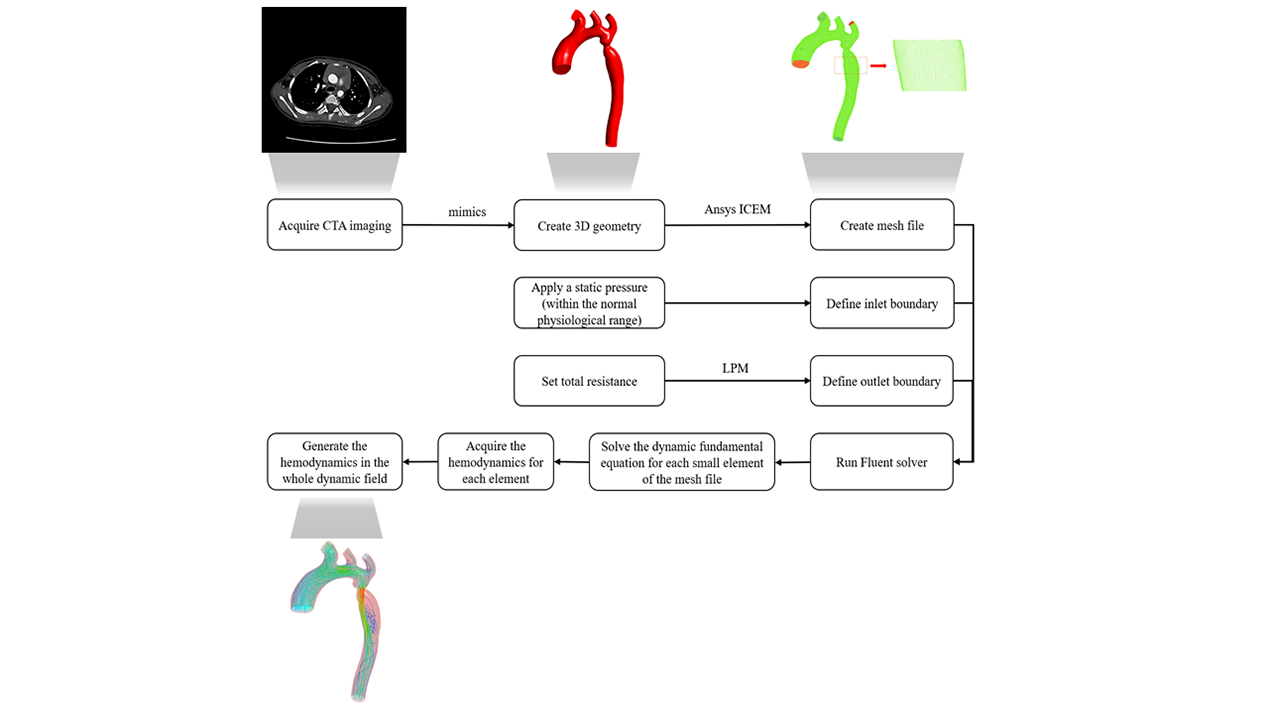


We used CTA imaging to generate a mesh file consisting of many discrete small mesh elements. The boundary conditions and the mesh file were imported into the Fluent software to solve the fundamental dynamics equation and acquire the hemodynamics of each discrete mesh element. Fluent was then also used to integrate the discrete elements into a complete dynamic field and acquire the hemodynamics for the whole field.

Online Figure 3 Results of the consistency test performed on the reconstruction and anatomic information


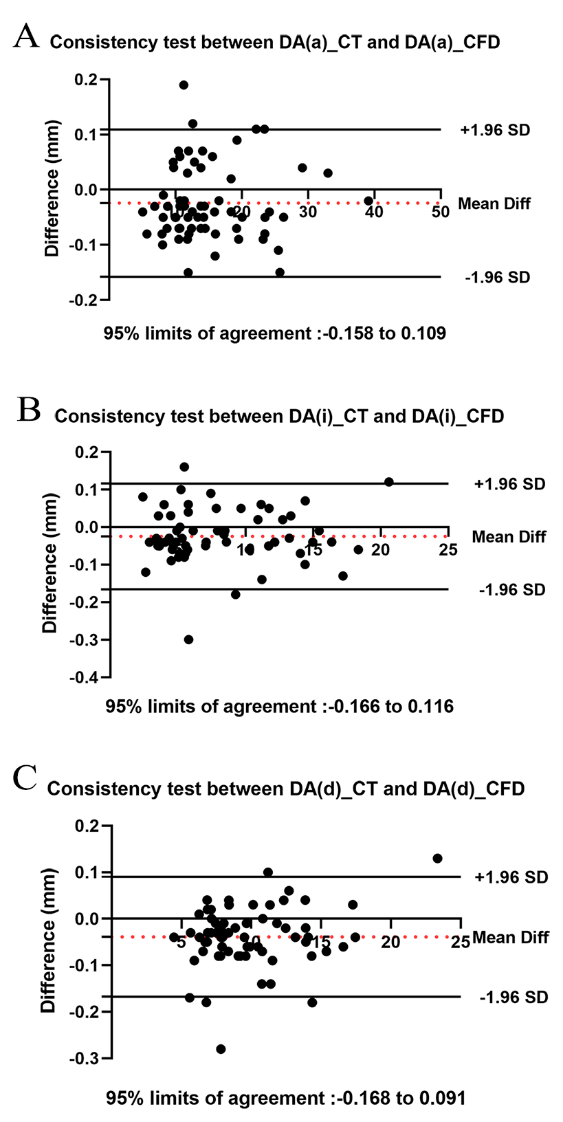


DA(a), DA(i), and DA(d) represent the diameters of the ascending aorta, aortic isthmus, and descending aorta, respectively. D_CT_ and D_CFD_ are the diameters measured through the CT workstation and the reconstructed CFD models, respectively. Mean Diff indicates the average difference among the diameters measured through the CT workstation and the reconstructed CFD models.
